# Mr. Wilkinson on the Air Bladder of Fish

**Published:** 1799-11

**Authors:** C. Wilkinson

**Affiliations:** Leicester Street, Leicester Square


GENTLEMEN,

IN Duncan's Annals of Medicine for the laſt year, is a paper by Mr. C. FISCHER, on the air bladder of a fiſh; his obſervations gave riſe to the following, which, if worthy of inſertion in your Journal, will be deemed an honour,

By your moſt obedient,

*Leiceſter Street, Leiceſter Square.* C. WILKINSON.

FISCHER ſuppoſes that this organ is deſtined to decompoſe water for the purpoſe of affording oxygen to the animal. If the oxygen is given out, only hydrogen ought to be found in the bladder: according to FISCHER, azotic and carbonic acid gas were more frequently diſcovered. To aſcertain this, I tried the following experiments.

As trout poſſeſs a larger air bladder, in proportion to their ſize, than any other fiſh, I procured ſome of their bladders, as freſh as poſſible; I opened them ſo as to let the air enter into my eudiometer. In making uſe of the nitrous teſt, the ſame diminution took place as in atmoſpheric air.

I opened another bladder under my receiver, expoſing the gas to lime water; no cretaceous precipitation enſued, which would neceſſarily, had it been carbonic acid gas.

In a ſmall glaſs exploding veſſel, I opened another, then mixed with it the proportionate part of hydrogen; on paſſing the electrical ſpark, the ſame exploſion took place as is obſerved with hydrogen and atmoſpheric air.

I took particular care that the bladders were opened before the contained air could have undergone any change.

It is ſurpriſing that bladders filled with any kind of gas, placed in a fura surrounding medium of a different gas, will soon lose its own, and imbibe the surrounding.

I filled a bladder with hydrogen gas for the space of two hours; the gas did not appear to undergo any change; after three hours, the bladder increased insensibly in weight; on examining the air, it was found so much mixed with atmospheric air, as to be rendered explosible.

I placed a bladder distended with hydrogen, under a large receiver of carbonic acid gas; after six hours the bladder was nearly half filled with the carbonic acid gas.

These experiments will induce one to believe that the bladder is so pervious, as to let the contained air gradually escape; this would not appear so surprising, were it not that the bladder remains equally distended, mixed with air of the surrounding medium, so that the bladder imbibes a particle of air for every particle it evolves; thus imperceptibly changing till an equilibrium takes place.

From these circumstances, it requires the utmost caution to make any inference from experiments on airs, confined in bladders for any time.

The bladders appear to answer no other purpose than to regulate the buoyancy of the fish.

The bladder is composed of two portions or bags, one lying within the thorax, the other portion in the abdomen.

These portions communicate with each other; in the quiet state of the animal, neither of them are quite distended; the thoracic portion lying in a cavity arched by bone, no diminution takes place in the bulk of the thorax by any alteration of the thoracic vesicular portion.

The abdomen varies with the changes of its vesicular portion.

The animal when he wants to rise, compresses the thoracic portion; this determines the air into the abdominal portion, increases the bulk of the animal, and renders him specifically lighter than the water.

To effect this compression, the thoracic vesicular portion is surrounded with a strong muscular theca, which the abdominal portion has not.

I see no physiological purpose that could be answered, by supposing it to be destined to decompose water.

For every office of respiration the gills answer, and which are well known to correfpond to our lungs, as they feparate the air from the water.

Fifhes foon die when placed in diftilled water.

---

